# Case Report: Limb salvage in *Vibrio vulnificus* necrotizing fasciitis through sequential debridement and skin grafting

**DOI:** 10.3389/fsurg.2026.1944678

**Published:** 2026-10-21

**Authors:** Yang Chen, Mengjing Xu, Shukai Zheng, Liyun Chen, Zijian Huang, Weiping Zeng, Yang Hao, Shijie Tang, Wancong Zhang

**Affiliations:** 1 Department of Plastic Surgery and Burns Center, Second Affiliated Hospital, Shantou University Medical College, Shantou, Guangdong, China; 2 Plastic Surgery Institute, Shantou University Medical College, Shantou, Guangdong, China; 3 Shantou Plastic Surgery Clinical Research Center, Shantou, Guangdong, China; 4 Research Center of Translational Medicine, Second Affiliated Hospital, Shantou University Medical College, Shantou, Guangdong, China

**Keywords:** case report, limb salvage, necrotizing fasciitis, sequential debridement, skin grafting, vacuum sealing drainage, *Vibrio vulnificus*

## Abstract

**Background:**

*Vibrio vulnificus* (*V*. *vulnificus*) is a highly aggressive pathogen that causes rapidly progressing necrotizing fasciitis and septic shock, frequently leading to limb amputation or death. Limb salvage in this setting remains challenging, particularly in diabetic patients.

**Case presentation:**

A 72-year-old diabetic male developed *V*. *vulnificus* necrotizing fasciitis of the left upper limb after handling marine fish, presenting with local erythema, swelling, warmth, pain, and septic shock. He underwent five sequential debridement procedures combined with vacuum sealing drainage (VSD), followed by artificial dermis application and split-thickness skin grafting, ultimately achieving successful limb salvage.

**Conclusion:**

In this patient, serial reassessment, staged debridement with VSD, and delayed wound reconstruction were associated with infection control and limb preservation. This case may inform the multidisciplinary management of severe *V. vulnificus* necrotizing fasciitis.

## Introduction

*Vibrio vulnificus* (*V*. *vulnificus*), a halophilic and thermophilic rod-shaped bacterium, is commonly found in warm coastal waters and estuaries (1). Recently, the incidence and geographic range of *V*. *vulnificus* infections have expanded due to global warming and an increase in extreme weather events (2). *V*. *vulnificus* can infect humans through contaminated seafood consumption or wound exposure to seawater or seafood (3). Infections can rapidly progress to necrotizing fasciitis and septicemia, particularly in patients with diabetes or chronic liver disease (4), with mortality rates reaching 50% and approaching 100% when treatment is delayed beyond 72 h (5, 6).

The subtle early symptoms and rapid progression of *V. vulnificus* infections to fatality pose significant clinical challenges. In this study, we describe successful limb salvage in a patient with severe *V*. *vulnificus* infection complicated by septic shock and necrotizing fasciitis, to illustrate the decision-making process behind a sequential surgical approach.

## Case presentation

A 72-year-old male with a 20-year history of diabetes and chronic alcohol use developed swelling, pain in the left upper limb, fever (38.5℃), and transient loss of consciousness one day after handling fresh marine fish, without obvious penetrating trauma. At emergency-department presentation, he was hypotensive [blood pressure (BP), 73/54 mmHg] and hypoxemic (oxygen saturation, 88%). Initial laboratory results showed a white blood cell (WBC) count of 6.1 × 10⁹/L, neutrophils (NE) of 81.4%, C-reactive protein (CRP) of 24.62 mg/L, and procalcitonin (PCT) of 16.7 ng/mL (Supplementary Table S1). After norepinephrine support, the patient was diagnosed with septic shock and admitted to the emergency intensive care unit (EICU). Initial resuscitative measures included endotracheal intubation under sedation, fluid resuscitation, and empiric antibiotics (imipenem-cilastatin 1 g intravenously every 8 h).

On the following day, the patient deteriorated clinically, presenting with persistent fever, requiring high-dose vasopressors for BP maintenance. Labs showed further elevation: WBC 10.2 × 10^9^/L, NE 94.4%, CRP 351 mg/L, PCT >100 ng/mL, interleukin-6 > 5,000 pg/mL. Treatment intensified with norepinephrine and epinephrine for BP support, and vancomycin (0.5 g intravenously every 12 h) was added for broader antimicrobial coverage. Plastic surgeons were consulted and observed significant swelling of the dorsum of the left hand and forearm, with violaceous-to-black skin discoloration. The skin and subcutaneous tissue were edematous, with scattered tense bullae (Figure 1A). Further medical history revealed his contact with seafood prior to illness, suggesting *V*. *vulnificus* infection. Subsequently, five surgeries, involving debridement with vacuum sealing drainage (VSD), were performed.

**Figure 1:**
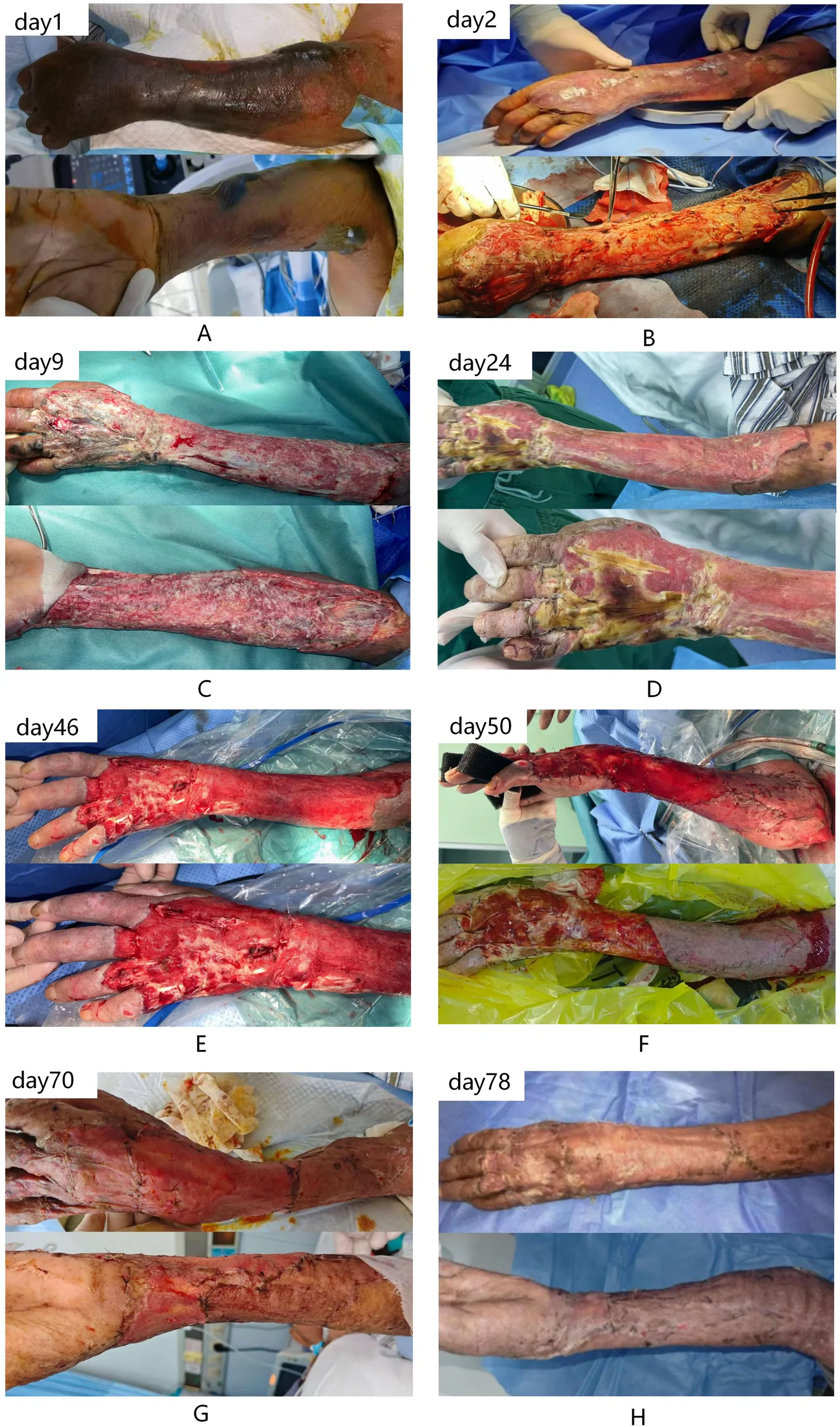
Wound treatment for *Vibrio vulnificus* infection: A visual guide to sequential management. **(A)** Significant swelling and blackening of the left upper limb with hemorrhagic bullae on admission. **(B)** During the first debridement, liquefied necrotic fat was found after incising the necrotic skin. **(C)** During the second debridement, an amount of yellowish-white slough covering the wound surface was observed. **(D)** During the third debridement, slough decreased. **(E)** During the fifth debridement, minimal slough was observed, with healthy granulation tissue and exposed tendons. **(F)** First-stage artificial dermis and autograft skin transplantation performed based on wound conditions. **(G)** Wound healing after the second split-thickness skin grafting. **(H)** Wound appearance after suture removal.

The first debridement (day 2) was performed as an emergency exploration triggered by rapid systemic deterioration, progressive skin changes, and a Laboratory Risk Indicator for Necrotizing Fasciitis (LRINEC) score of 8 (Supplementary Table S1). Intraoperative exploration revealed necrotic skin with blackish-brown dermis, early patchy fascial necrosis interspersed with viable tissue, purulent exudate tracking along fascial planes, and a positive finger test, while the underlying muscle appeared grossly viable (Figure 1B). After necrosectomy and irrigation, VSD was applied at −20 kPa in continuous mode. Throughout the debridement phase, the VSD dressing was removed after 7 days to assess the wound; thereafter, the wound was disinfected and redressed every 2 days until the next operation when VSD was reapplied, and this interval was not considered final discontinuation of VSD. Postoperatively, even when surgery was delayed until 36 h after symptom onset, the patient demonstrated significant clinical improvement with hemodynamic stabilization and decreases in CRP (to 141 mg/L) and PCT (to 8.88 ng/mL) (Figure 2). Admission blood cultures confirmed *V*. *vulnificus* approximately 72 h later, and subsequent susceptibility testing showed sensitivity to all tested antibiotics, prompting adjustment to levofloxacin (0.5 g intravenously once daily) and imipenem-cilastatin.

**Figure 2:**
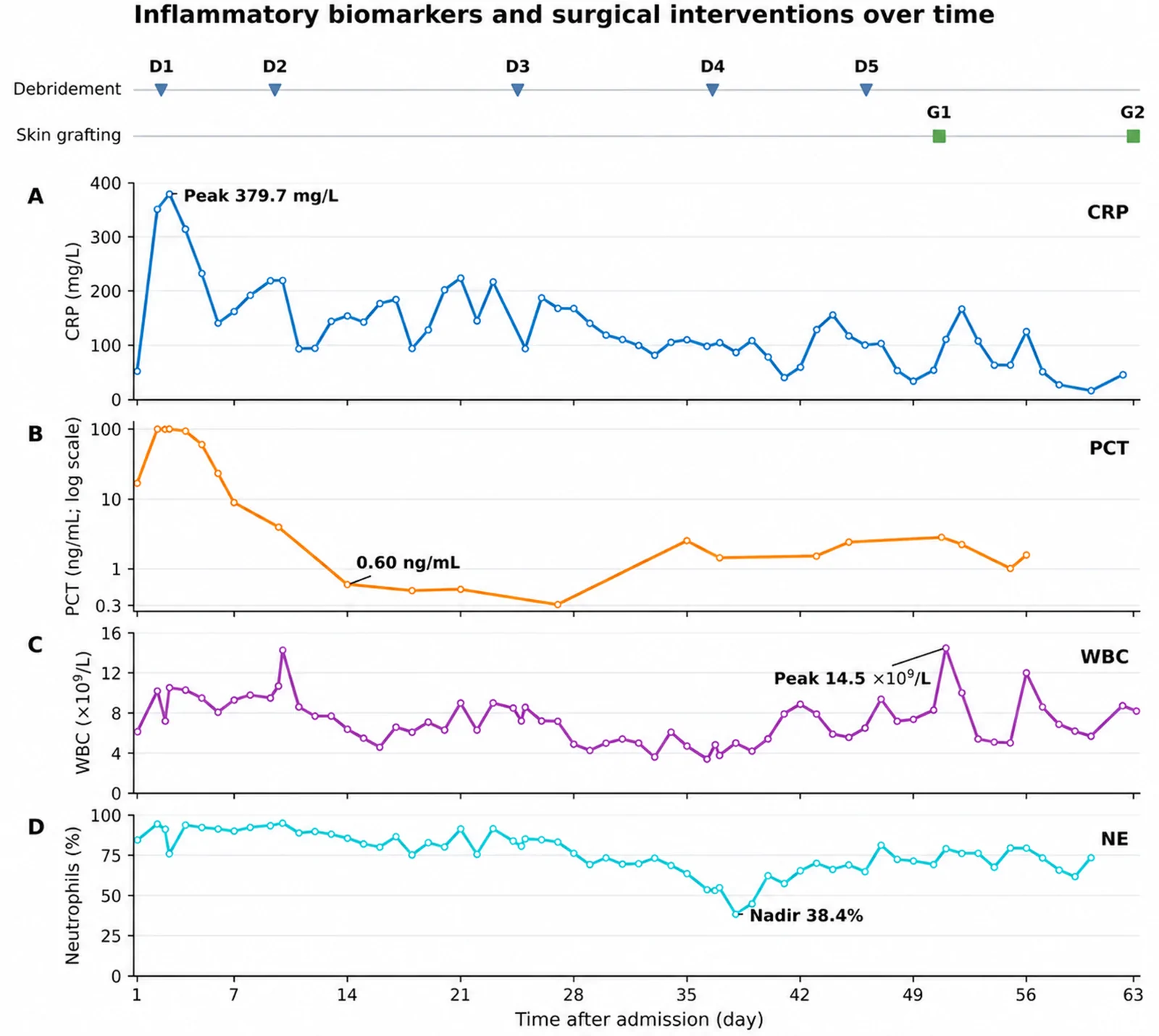
Inflammatory biomarkers and surgical interventions over time. Separate panels show **(A)** C-reactive protein (CRP, mg/L), **(B)** procalcitonin (PCT, ng/mL; logarithmic scale), **(C)** white blood cell count (WBC, 10^9^/L), and **(D)** neutrophil percentage (NE, %). Data points indicate available measurements, and lines connect successive measurements. D1–D5 denote debridement procedures on days 2, 9, 24, 36, and 46, respectively; G1–G2 denote skin-grafting procedures on days 50 and 63, respectively. Only selected clinically relevant values are labeled. The figure shows temporal associations and does not establish a causal effect of any intervention.

The second debridement (day 9) was prompted by persistent ill-defined necrotic margins and sustained elevation of inflammatory markers, and intraoperatively the ill-defined necrotic margins were addressed (Figure 1C). Thereafter, inflammatory-marker levels generally declined and vasopressor support was progressively reduced.

The third debridement (day 24) was performed after recurrent fluctuations in inflammatory markers and progressive demarcation of the necrotic margins (Figure 1D), which permitted more selective excision. The 15-day interval from the second procedure reflected the evolving demarcation rather than a planned delay in source control. Postoperatively, systemic inflammatory markers had returned to near-normal levels (Figure 2), and both blood and wound cultures showed no growth, indicating that sepsis was under control. Accordingly, antimicrobial therapy was de-escalated after this procedure and transitioned to cefoperazone (1 g intravenously every 8 h) for continued coverage. Subsequently, the patient gradually regained consciousness.

The fourth and fifth procedures were performed primarily for wound-bed preparation on day 36 and day 46, respectively. Progressive necrosis and purulence were no longer observed, and the wound showed increasing healthy granulation tissue (Figure 1E). From this stage onward, the VSD pressure was reduced to −16.6 to −10.6 kPa to promote granulation and protect the exposed tendons.

On day 50, Lando® bilayer artificial dermis (Shenzhen Landu Biomaterials Co., Ltd), comprising an outer silicone membrane and an inner collagen scaffold, was used to cover the exposed flexor tendons, while the first autologous split-thickness skin grafting (0.3 mm thickness, harvested from the lateral thigh using a pneumatic dermatome) was applied to the remaining granulating wound areas (Figure 1F). The artificial dermis was secured to the wound margins with absorbable sutures, and the autologous skin grafts were covered with petrolatum gauze and immobilized with VSD. On day 63, the silicone layer of the artificial dermis was removed and a second autologous split-thickness skin grafting was applied to the residual defects (Figure 1G). After the final grafting, VSD was discontinued once graft take exceeding 95% was confirmed and the wound could be managed with routine dressings.

Following transfer to the Department of Plastic Surgery on day 65 for continued management, the patient was discharged on day 74 and returned for suture removal on day 78 (Figure 1H). At three-month follow-up, he expressed gratitude for limb salvage. He acknowledged the physical and psychological burden of prolonged hospitalization and multiple surgeries, but considered these sacrifices worthwhile given the severity of infection and tissue loss. Hand function improved substantially: he could eat, write, and grasp large objects independently, although fine motor tasks remained mildly difficult. Examination showed active finger and wrist motion without tendon adhesion or contracture, while sensation was slightly diminished from baseline. He stated he would continue wearing protective gloves when handling raw seafood.

## Discussion

Early diagnosis of *V*. *vulnificus* infection is important for survival and possible limb salvage. The 2025 Expert Consensus introduced the “Double-SR” tool to aid in early suspicion among high-risk populations (7). However, this patient presented with shock and impaired consciousness, and seafood exposure history was unobtainable for 30 h, impeding early recognition. While microbiological culture remains the diagnostic gold standard as per the consensus, it took approximately 72 h from admission for blood cultures to confirm *V*. *vulnificus* in this case, posing a risk of diagnostic delay. Although emerging rapid diagnostics such as polymerase chain reaction and metagenomic next-generation sequencing hold promise, their clinical utility remains constrained by issues of cost, accessibility, and lack of standardization (8, 9). Thus, early clinical recognition based on evolving skin signs and systemic severity remains paramount when disease progression outpaces laboratory confirmation.

Despite the undetermined etiology, the emergency transfer to the EICU provided a critical resuscitation window. Alongside supportive care, the antimicrobial strategy aligned with the 2025 Chinese Expert Consensus (7), which recommends early combined full-dose therapy for severe *V*. *vulnificus* sepsi. However, antimicrobial therapy alone is often insufficient to control *V*. *vulnificus* infections (10), likely due to poor penetration of antibiotics into the necrotic core of the infection (11). In this patient, rapidly progressive skin changes and a LRINEC score rising from 4 on day 1 to 8 on day 2 (Supplementary Table S1) raised suspicion of necrotizing fasciitis and jointly indicated the need for urgent surgical debridement, underscoring the importance of prompt surgical consultation during EICU resuscitation (12, 13). The consensus advocates for prompt and, when necessary, multiple surgical debridements (7). In this case, the decision to adopt a multistage surgical approach was based on clinical considerations. Holistically, the patient's advanced age, poor general condition, and multiple comorbidities prolonged the course of infection control. Locally, the indistinct wound margins during the early infectious phase made it impossible to achieve complete excision in a single procedure. From a mechanistic perspective, *V*. *vulnificus* has been proposed to utilize multiple virulence factors to release toxic substances and promote bacterial colonization at the primary infection site (14). These properties may explain why single debridement often fails to achieve complete pathogen clearance. Taken together, the patient underwent five sequential debridements. The first three procedures addressed suspected or newly demarcated necrotic tissue, whereas the fourth and fifth procedures focused on preparing the wound bed after systemic stabilization. Complementing this surgical approach, continuous VSD therapy may have contributed to infection control by draining virulence factors and inflammatory mediators, thereby potentially reducing toxin accumulation and limiting infection spread. Following this sequential approach, the patient achieved hemodynamic stabilization, normalization of inflammatory markers, and formation of clean granulation tissue, ultimately contributing to limb preservation. However, given the concurrent multimodal treatment, this improvement cannot be attributed to surgery alone.

Reports of successful limb salvage in upper-extremity *V*. *vulnificus* necrotizing fasciitis remain scarce, possibly due to the small compartment volume, thin fascial barriers, and rich vascular supply of the forearm and hand, which predispose to rapid cross-compartmental spread and early systemic toxicity. In a Chinese case series of 11 patients with predominantly hand involvement, Lin et al. documented 4 amputations and 2 deaths, emphasizing that successful salvage is not always achievable even with surgical treatment (15). Among sporadic salvage reports, most describe a single debridement or flap-based reconstruction (16), with little detail about the timing or rationale for staged surgery. In this patient, repeated assessment and selective excision of clearly nonviable tissue, with preservation of grossly viable functional structures, were associated with infection control and limb preservation. The timing and extent of excision must be individualized according to infection progression, operative findings, systemic stability, and the feasibility of reconstruction.

Once the infection was controlled, treatment shifted to reconstruction. Current guidance on *V. vulnificus* infection does not specify how residual defects should be managed (7), although bilayer artificial dermis and autologous skin grafting have been used for wounds with exposed tendon or bone (17, 18). The 2019 Expert Consensus on Bilayer Artificial Dermis highlights that the silicone membrane prevents bacterial invasion, while its collagen scaffold promotes vascularization and granulation growth, accelerating healing and inhibiting scar formation (19). In this patient, artificial dermis provided temporary coverage of the exposed flexor tendons to prevent adhesion and necrosis, followed by staged split-thickness skin grafting for wound closure. VSD may have helped stabilize the grafts, control fluid accumulation, and facilitate neovascularization of the artificial dermis. Wound closure and limb preservation were maintained at 3 months, but longer follow-up is needed to assess tendon function, scar maturation, and sustained functional recovery.

This report is limited by its single-patient design and the absence of a comparator. Given that resuscitation, several antimicrobial regimens, sequential debridement with VSD were used concurrently, the outcome cannot be attributed to any one intervention. Functional recovery was assessed by patient report and clinical examination rather than validated tools, and follow-up was limited to three months. Consequently, the report is descriptive and cannot support comparative claims about treatment efficacy. Its purpose is instead to document the timing, rationale, and changing objectives of the staged interventions used in this uncommon and rapidly progressive infection.

In conclusion, this patient with severe *V. vulnificus* necrotizing fasciitis and septic shock achieved infection control and limb preservation through a staged surgical approach. The strategy began with emergency source control, progressed to repeated assessments and selective debridement as necrosis demarcated, and culminated in delayed reconstruction. This case suggests that, in carefully selected patients, adapting the surgical objective to the clinical stage may support limb preservation without compromising source control.

## Data Availability

The original contributions presented in the study are included in the article/Supplementary Material, further inquiries can be directed to the corresponding author.
