# Supplementary material for: Case Report: Limb salvage in Vibrio vulnificus necrotizing fasciitis through sequential debridement and skin grafting
Source: Front Surg. 2026 Oct 21;13:1944678. doi: 10.3389/fsurg.2026.1944678 (PMC13731324; doi:10.3389/fsurg.2026.1944678)
Supplement: Supplementary file 1 [file Table1.docx]

| **Parameter** | **Day 1 (admission)** | **Day 2 (pre-first debridement)** | **Day-2 LRINEC points** |
| --- | --- | --- | --- |
| CRP (mg/L) | 24.62 | 351 | 4 |
| WBC (×10⁹/L) | 6.1 | 10.2 | 0 |
| Hemoglobin (g/L) | 118 | 125 | 1 |
| Sodium (mmol/L) | 137.1 | 136 | 0 |
| Creatinine (μmol/L) | 173 | 210.8 | 2 |
| Glucose (mmol/L) | 14.4 | 27.1 | 1 |
| PCT (ng/mL) | 16.7 | >100 | Not included |
| NE (%) | 81.4 | 94.4 | Not included |
| Interleukin-6 (pg/mL) | >5,000 | >5,000 | Not included |
| Platelet count (×10⁹/L) | 145 | 120 | Not included |
| SAA (μg/mL) | 172 | - | Not included |
| Lactate (mmol/L) | 4.09 | - | Not included |
| Potassium (mmol/L) | 3.32 | 3.53 | Not included |
| Other laboratory findings | Troponin slightly elevated; CK and CK-MB within normal ranges; liver function and coagulation parameters normal | CK 257 U/L; prolonged coagulation time; fibrinogen and D-dimer slightly elevated | Not included |
| Total LRINEC score | 4 | 8 | 8 |

Supplementary Table S1. Admission and pre-first-debridement laboratory parameters and LRINEC score. Abbreviations: CK, creatine kinase; CK-MB, creatine kinase-MB; CRP, C-reactive protein; LRINEC, Laboratory Risk Indicator for Necrotizing Fasciitis; NE, neutrophil percentage; PCT, procalcitonin; SAA, serum amyloid A; WBC, white blood cell count. The LRINEC score was calculated from CRP, WBC, hemoglobin, sodium, creatinine, and glucose values.
